# SO_2_ Management and Yeast Inoculation Strategies (NoSO_2_-Spont, NoSO_2_Sc, SO_2_Sc) During Fermentation Shape the Chemical, Polyphenolic, Microbiological, and Sensory Profiles of ‘Solaris’ White Wine

**DOI:** 10.3390/molecules31081344

**Published:** 2026-04-19

**Authors:** Magdalena Błaszak, Ireneusz Ochmian, Ireneusz Kapusta, Sabina Lachowicz-Wiśniewska

**Affiliations:** 1 Department of Bioengineering, West Pomeranian University of Technology in Szczecin, Słowackiego 17 Street, 71-434 Szczecin, Poland; magdalena.blaszak@zut.edu.pl; 2 Department of Horticulture, West Pomeranian University of Technology Szczecin, Słowackiego 17 Street, 71-434 Szczecin, Poland; 3 Department of Food Technology and Human Nutrition, Rzeszów University, Zelwerowicza 4 Street, 35-601 Rzeszów, Poland; ikapusta@ur.edu.pl; 4 Department of Medical and Health Sciences, University of Kalisz, W. Bogusławskiego Square 2, 62-800 Kalisz, Poland; 5 Department of Biotechnology and Food Analysis, Wroclaw University of Economics and Business, 53-345 Wroclaw, Poland

**Keywords:** spontaneous fermentation, sulphur dioxide (SO_2_) management, *Saccharomyces cerevisiae*, polyphenolic profile, volatile acidity

## Abstract

Consumer interest in low-SO_2_ white wines is increasing; however, such approaches may reduce compositional and sensory predictability. This study evaluates how three fermentation strategies—SO_2_ addition and *Saccharomyces cerevisiae* ES181 inoculation (SO_2_Sc), spontaneous fermentation (NoSO_2_-Spont), and inoculation with *S. cerevisiae* ES181 without SO_2_ addition (NoSO_2_Sc)—shape the chemical profile, polyphenolic composition, colour, microbiological status, and sensory perception of ‘Solaris’ wines relative to the must (reference). A single batch of ‘Solaris’ must (one press run) was split into three variants and fermented under identical temperature conditions (12 ± 0.5 °C), followed by cool ageing and natural sedimentation prior to bottling. Basic oenological parameters, selected fermentation by-products, viable yeast counts, CIE Lab colour, targeted polyphenolics (phenolic acids, flavonols, flavan-3-ols, and stilbenes), PCA of by-products, and blind sensory evaluation were assessed. The NoSO_2_-Spont variant showed reduced fermentation completeness (higher residual sugars and lower ethanol) and the highest volatile acidity, together with elevated glycerol and several higher alcohols, and received the lowest sensory ratings. The SO_2_Sc variant yielded the most controlled outcome, with the lowest volatile acidity, the brightest colour (higher L*, lower b*), and the highest sensory acceptance. The NoSO_2_Sc variant produced intermediate sensory scores and a higher total phenolic content; however, volatile acidity remained high and viable yeast counts were the greatest, indicating increased susceptibility to microbiological activity during extended pre-bottling handling. Overall, the SO_2_Sc strategy provides the greatest chemical stability and sensory acceptance, whereas low-SO_2_ regimes require a hurdle approach (oxygen control, residual sugar management, hygiene, and stabilisation) to limit spoilage development and post-bottling refermentation.

## 1. Introduction

Consumer interest in wines produced with reduced technological intervention has increased in parallel with a broader preference for organic and minimally processed foods. In the wine sector, this trend is commonly associated with production approaches based on limited additive input, spontaneous fermentation, and restricted use of sulphur dioxide (SO_2_). As a result, scientific and technological interest has intensified in vinification strategies that reduce preservative use while maintaining chemical and microbiological stability and ensuring acceptable sensory quality [1,2].

Among oenological preservatives, sulphiting remains the most widely applied practice in must and wine production. Sulphur dioxide and its authorised salts (e.g., potassium metabisulphite) are effective antimicrobial and antioxidant agents and are permitted within regulatory limits [3,4]. Nevertheless, the use of sulphites continues to attract attention due to adverse reactions reported in sensitive individuals, including bronchoconstriction and asthma exacerbation, with an estimated prevalence of around 1% in the general population [1]. Beyond safety considerations, sulphitation may influence wine composition by modulating oxidation–reduction reactions and microbial activity, thereby affecting aroma development and the fate of phenolic constituents that contribute to colour, mouthfeel, and bioactivity [2]. In line with this, recent research increasingly addresses no-added-SO_2_ or strongly reduced-SO_2_ protocols, indicating that technological feasibility and chemical stability can be achieved when fermentation and oxygen exposure are managed under controlled winery conditions [5].

In contrast to inoculated, sulphited fermentations, spontaneous fermentations are initiated and driven by the native microbiota associated with grape surfaces and winery environments. These communities typically include non-*Saccharomyces* yeasts (e.g., genera: *Hanseniaspora*, *Metschnikowia*, *Pichia*, *Torulaspora*, and *Candida*) and bacteria relevant to winemaking (e.g., lactic acid bacteria such as *Oenococcus* and *Lactobacillus* genera, and acetic acid bacteria such as *Acetobacter* and *Gluconobacter*) [6]. Microbial succession during spontaneous fermentation can increase compositional and sensory complexity through the formation of higher alcohols, esters, aldehydes, organic acids, and volatile phenols; however, the direction and magnitude of these changes are variable and depend on must composition, fermentation conditions, and the initial microbial consortium [7,8]. Contemporary studies using culture-independent and omics-based approaches further confirm that microbial dynamics during natural fermentation can be strongly linked to changes in chemical profiles and fermentation behaviour, reinforcing the current relevance of microbiome-informed vinification [9].

A major limitation of fully spontaneous vinifications is the elevated risk of technological failures and quality defects, including excessive volatile acidity, stuck or sluggish fermentations, oxidative deviations, and the formation of undesirable compounds such as ethyl acetate, acetoin, and volatile phenols associated with spoilage microorganisms (including *Brettanomyces* spp.) [7,10,11,12]. From an industrial perspective, such variability can translate into inconsistent sensory performance and shortened shelf-life, which remains a key barrier to the wider adoption of low-SO_2_ winemaking, despite its appeal to niche consumers and its alignment with sustainability narratives [12,13]. In addition, unmanaged microbiota may elevate safety concerns related to the accumulation of certain metabolites, including biogenic amines, under conditions that favour uncontrolled bacterial growth [14]. Notably, very recent evidence demonstrates that specific process decisions during alcoholic and malolactic fermentation, together with must clarification, can significantly modulate biogenic amine formation, highlighting the need for controlled strategies when sulphite inputs are reduced [15].

These considerations illustrate a practical trade-off between two extremes: (I) conventional vinification relying on sulphitation to support predictable fermentation kinetics and stability, and (II) spontaneous, low-intervention vinification that may enhance microbially driven complexity but carries an increased risk of defects and instability. A promising direction is therefore an intermediate strategy that reduces or eliminates sulphiting while retaining technological control over fermentation. One such strategy is the inoculation of non-sulphited must with robust commercial *Saccharomyces cerevisiae* strains (including *S. cerevisiae* ES181), selected for reliable fermentation performance and tolerance to stress conditions. In parallel, bioprotection based on selected non-*Saccharomyces* yeasts (e.g., *Metschnikowia pulcherrima*) has been actively explored as a low-input approach to constrain spoilage microbiota and reduce SO_2_ demand while maintaining volatile composition, typically via sequential or mixed fermentations with *S. cerevisiae* [16]. Collectively, these recent developments support the concept that SO_2_ reduction can be achieved not only through chemical substitution, but also through targeted microbiological management.

For the cool-climate cultivar ‘Solaris’, this issue is particularly relevant because the variety has already been investigated in several European wine-producing regions, demonstrating both broad adaptation to northern growing conditions and considerable flexibility in winemaking. In addition to vinification studies, recent research has shown that ‘Solaris’ has also been evaluated in relation to agronomic and quality-related production factors. Under north-western Polish conditions, multi-year plant protection with copper-based pesticides was found to influence heavy metal accumulation patterns in vineyard soil and plant organs, highlighting that vineyard protection practices may contribute to the environmental and compositional context in which disease-resistant grape cultivars such as ‘Solaris’ are produced [17]. Moreover, juice obtained from organically and conventionally grown ‘Solaris’ grapes differed in antioxidant, nutritional, microbiological, and health-safety attributes, indicating that differences in the grape production system may also influence the compositional quality of the raw material entering vinification [18]. In Denmark, monovarietal ‘Solaris’ wines were shown to differ markedly in volatile and non-volatile composition as well as in sensory profile, indicating that this cultivar can yield wines with distinct chemical and sensory characteristics under practical production conditions [19]. In a subsequent Danish study, different pre-fermentation strategies applied to ‘Solaris’ grapes, including direct pressing, whole-cluster pressing, cold maceration, and skin fermentation, significantly modified volatile composition, polyphenol levels, and sensory expression, confirming that ‘Solaris’ is highly responsive to vinification decisions [20]. Likewise, research on Swedish ‘Solaris’ wines reveals a broad sensory space and limited agreement on a single “typical” style, suggesting that this cultivar can be shaped into diverse wine styles depending on regional conditions and oenological choices [21]. More recently, ‘Solaris’ has also been included among disease-resistant white cultivars assessed in South Tyrol, further supporting its growing relevance in contemporary European winemaking systems [22].

Despite growing interest in sulphite reduction, only a limited number of controlled side-by-side studies have simultaneously compared the effects of sulphitation and yeast inoculation on fermentation behaviour, wine composition, and sensory properties under otherwise matched winemaking conditions [23,24,25]. In particular, evidence remains limited regarding whether a “no-SO_2_ but inoculated” protocol can consistently provide a profile closer to spontaneous wines in terms of complexity and phenolic expression, while approaching conventional wines in terms of stability and freedom from major defects.

Accordingly, this study evaluates an intermediate vinification strategy based on non-sulphited must inoculated with *S. cerevisiae* ES181, benchmarked against a sulphited inoculated control and a fully spontaneous fermentation. We hypothesise that inoculation without sulphiting will (I) reduce spoilage-associated metabolites and technological deviations relative to spontaneous fermentation, while (II) preserving favourable chemical and sensory attributes and maintaining a more “low-intervention” character than sulphited controls. By addressing the combined expectations of safety, stability, and product authenticity, this work contributes to the development of scalable, low-SO_2_ winemaking protocols relevant to both research and commercial production.

## 2. Results and Discussion

### 2.1. Yeasts in Must and Wine

The results indicate that SO_2_ addition and the inoculation scheme applied at the onset of fermentation significantly affect the number of viable yeasts quantified in the musts and finished wines. Yeast counts are presented as means and expressed as log10 CFU/mL, as shown in Table 1. In addition, selected original non-log-transformed values and percentage comparisons of yeast abundance before and after fermentation are included in the text to facilitate interpretation of the biological magnitude of the observed changes.

To interpret the final counts correctly, it is essential to consider the initial yeast load in the must before fermentation, which differed among the variants due to the presence of autochthonous microbiota and the inoculation strategy applied. In the spontaneous variant (NoSO_2_-Spont), indigenous yeasts were naturally present in the must at an average of 4.88 log10 CFU/mL prior to fermentation. In the inoculated variant (NoSO_2_Sc), the same must containing indigenous yeasts was inoculated at the onset of fermentation with a commercial strain of *S. cerevisiae* ES181. In this variant, the initial yeast population was the highest, reaching approximately 5.5 log10 CFU/mL. In the sulphited and inoculated variant (SO_2_Sc), the must was first sulphited and then inoculated with *S. cerevisiae* ES181 at the same dose as in NoSO_2_Sc. In this case, SO_2_ most likely reduced the contribution of indigenous yeasts present in the must before inoculation, and the total yeast count measured in this variant was approximately 5.4 log10 CFU/mL. This indicates that the contribution and early survival of autochthonous yeasts before fermentation were likely substantially reduced by SO_2_ treatment.

After fermentation, the yeast count decreased significantly in all variants. Viable yeast counts in the three wine variants differed significantly from one another. The lowest yeast population was observed in the spontaneous wine, in which only 1.48 log10 CFU/mL viable cells were detected at the time of analysis. This value corresponds to several dozen CFU per mL of wine (original non-log-transformed data). Notably, this very low final count occurred despite the relatively high initial abundance of indigenous yeasts in the must (4.88 log10 CFU/mL), suggesting a pronounced decline in survival and/or culturability during fermentation and subsequent ageing.

To further characterise the surviving culturable yeast population in this spontaneous variant, representative isolates recovered from the NoSO_2_-Spont wine were subjected to sequence-based molecular identification. This analysis revealed only two sequence types of *S. cerevisiae* (GenBank accession numbers: MF169658.1 and CP006466.1). This finding suggests that spontaneous fermentation followed by ageing may impose strong selective pressure, ultimately favouring only a limited number of *S. cerevisiae* variants and/or substantially reducing the culturability of other yeasts. Although *S. cerevisiae* usually represents only a minor proportion of the initial environmental yeast population, when present in the must it may assume a dominant role during fermentation because of its greater tolerance to increasing ethanol concentrations. This behaviour is consistent with the broader concept that autochthonous microbiota may increase diversity at the early stages, whereas the final microbiological outcome depends strongly on must and wine conditions as well as process management [26].

By contrast, in the sulphited variant (SO_2_Sc), the mean yeast count in the finished wine was 3.63 log10 CFU/mL (i.e., several thousand CFU/mL, based on the original non-log-transformed data), indicating that SO_2_ addition combined with controlled inoculation supported a more predictable process and likely limited competition from autochthonous microbiota while supporting the dominance of the inoculated yeast strain. This observation is consistent with the well-established role of sulphur dioxide as a selective antimicrobial and antioxidant tool that facilitates fermentation management and post-fermentation stability while restricting the development of undesirable microbial consortia [27].

The highest viable yeast count was recorded in the NoSO_2_Sc variant, reaching 4.40 log10 CFU/mL (i.e., more than 25 thousand CFU/mL, based on the original non-log-transformed data). This result should not be interpreted solely as an effect of a less selective fermentation environment, because key determinants are the absence of SO_2_ and the initial microbiological background of the must: this variant began with an indigenous yeast load together with an additional inoculum dose, which may have favoured higher post-fermentation cell survival and/or regrowth during ageing.

All variants showed a statistically significant and clear reduction in yeast numbers. Final viable yeast counts ranged from several dozen CFU/mL (corresponding to approximately 99.9% reduction in the NoSO_2_-Spont variant) to approximately 7% of the initial level in NoSO_2_Sc. In the sulphited wine (SO_2_Sc), the final yeast count corresponds to approximately 2% of the initial level. These percentages are expressed relative to the initial yeast abundance (CFU/mL) measured before fermentation. From a practical perspective, such an outcome may increase the likelihood of microbiological instability and the risk of renewed fermentation (refermentation) if residual fermentable substrates remain or if storage conditions fluctuate—an issue repeatedly emphasised in low-SO_2_ winemaking [27].

These results should be considered within the current market and research context. In recent years, wines produced without SO_2_ addition and relying on spontaneous fermentation have become more visible and are often produced using spontaneous fermentation driven by autochthonous yeasts [12]. A substantial body of literature indicates that non-*Saccharomyces* yeasts can generate metabolites that modulate aroma, mouthfeel, and overall sensory complexity, particularly when applied in mixed or sequential fermentations with *Saccharomyces* [26]. At the same time, these effects are highly context-dependent: depending on the species/strain composition and the degree of process control, spontaneous fermentations may increase batch-to-batch variability and the probability of sensory deviations, including undesirable notes and elevated volatile acidity. More recent studies emphasise that the desired “complexity” is achieved more reliably when autochthonous or selected non-*Saccharomyces* yeasts are implemented in controlled mixed-culture strategies, rather than being left entirely unmanaged [28].

From the perspective of applied oenology, this helps to explain why strains conferring valued aroma–flavour attributes have long been selected for commercial use [29]. Conversely, a subset of consumers deliberately seeks unconventional and atypical sensory profiles, reinforcing the niche positioning of wines produced with minimal technological intervention. This niche positioning is also reflected in patterns of sensory acceptance reported for wines produced without conventional stabilisation, where sensory distinctiveness may be perceived either as an attractive signature or as a fault, depending on consumer expectations [14]. Therefore, linking our microbiological findings directly to sensory outcomes should be undertaken cautiously: higher or lower yeast viability in the final product does not automatically translate into better or worse sensory quality, but may instead indicate differences in process predictability and microbiological stability.

Finally, the question raised in the original text—whether the uniqueness of such wines may affect consumer health—should be addressed in an evidence-based manner and without speculation. The most defensible approach is to refer to food safety and quality assurance, rather than making direct health claims. In wines with reduced SO_2_ addition, the primary practical concern is typically increased susceptibility to spoilage or unintended microbial activity during storage, rather than any direct toxicological effect of sensory distinctiveness per se. Contemporary reviews consistently indicate that, although multiple alternatives can partially substitute for SO_2_ (e.g., chitosan-based approaches, DMDC, membrane filtration, UV, ultrasound, high-pressure processing, pulsed electric fields), none of these measures alone reproduce the full antimicrobial and antioxidant protection provided by sulphur dioxide. Consequently, when SO_2_ is reduced, hurdle strategies, together with stringent hygiene and rigorous process control, are recommended [27].

### 2.2. Basic Parameters of Must and Wines

In Table 2, the basic parameters of the must (baseline reference) and the wines produced under three fermentation variants are summarised: SO_2_Sc, NoSO_2_-Spont, and NoSO_2_Sc. Analyses were performed after completion of alcoholic fermentation, following natural sedimentation, and immediately prior to bottling (at 12 °C). Significant differences were observed in acidity-related parameters, the extent of sugar depletion, and indicators linked to potential microbiological stability.

The increase in pH observed in the non-sulphited variants is likely attributable to the concurrent action of several processes occurring during fermentation and subsequent sedimentation. First, under cool fermentation (12 °C) and lees ageing conducive to clarification, tartrate stabilisation may occur, with precipitation of tartrate ions as crystals (predominantly potassium bitartrate), thereby altering acid–base equilibria and potentially shifting pH independently of titratable acidity (TA), depending on the wine’s ionic composition and initial pH range [30,31]. Second, final pH and TA are influenced by the wine’s buffering chemistry: even with a similar total pool of titratable acids, differences in the proportion of organic acid salts and in cation content (particularly K^+^) may result in an increase in pH while TA remains comparable or changes in a different direction. Such a decoupling of pH and TA is widely described as an outcome of buffering capacity and ionic equilibria, particularly in white wines with heterogeneous acid profiles [32,33].

In addition, in variants without SO_2_, microbial activity affecting the acid balance is more likely—for example, the initiation of acid biotransformations and related microbial metabolic activity—which may lead to a perceptible softening of acidity alongside an incompletely predictable pH–TA relationship [34,35]. Practically, this means that TA alone is insufficient to assess microbiological risk: a higher pH in non-sulphited wines may increase susceptibility to microbial growth during the pre-bottling period and storage, even when TA remains relatively high.

Sugar parameters clearly reflected fermentation completeness across the variants. The must contained high concentrations of glucose and fructose (108 and 122 g/L, respectively), whereas after fermentation their levels decreased to values typical of dry wines. However, significant differences were evident for residual sugars: the NoSO_2_-Spont wine retained higher concentrations of glucose (2.4 g/L) and fructose (4.5 g/L) than the inoculated variants (NoSO_2_Sc and SO_2_Sc) (glucose 0.3–0.8 g/L; fructose 1.6–2.3 g/L). This outcome is consistent with reports that, in spontaneous fermentations, variable yeast succession (including contributions from non-*Saccharomyces* yeasts) may promote slower kinetics and a greater risk of incomplete sugar depletion, particularly in the terminal phase of the process when ethanol stress increases and nutrient limitations become more pronounced [26].

The effect of SO_2_ addition was clearly apparent in the SO_2_Sc variant, in which total SO_2_ reached 44 mg/L and free SO_2_ 27 mg/L (group b), whereas in the must as well as in the non-sulphited variants (NoSO_2_-Spont and NoSO_2_Sc) both SO_2_ fractions remained very low, at only 5–7 mg/L for total SO_2_ and 3 mg/L for free SO_2_. From a technological standpoint, this difference is highly relevant, because the antimicrobial and antioxidant action of SO_2_ depends not only on the total content, but primarily on the free fraction available in wine. Moreover, the inhibitory effectiveness of free SO_2_ against microbiota, particularly lactic acid bacteria, is strongly modulated by pH, which determines the proportion of molecular SO_2_, i.e., the most microbiologically active form. Consequently, the combination of higher pH and very low free SO_2_ in the non-sulphited variants may substantially reduce microbiological protection during maturation and immediately prior to bottling, despite small differences in total SO_2_. By contrast, the SO_2_Sc variant combined the highest free SO_2_ concentration with a comparatively lower pH than the non-sulphited wines, which would be expected to enhance the protective effect of sulphiting and improve microbiological stability in the late stages of vinification [34,36].

The profile of organic acids suggests the co-occurrence of chemical stabilisation phenomena and microbiologically mediated transformations. Tartaric acid was highest in the must (baseline reference) (4.5 g/L) and decreased in all wines, most markedly in the NoSO_2_-Spont variant (3.5 g/L), which is consistent with the typical precipitation of tartrate salts during fermentation and cool ageing. Malic acid was significantly lower in the SO_2_Sc wine (2.1 g/L) than in the must (2.5 g/L), whereas the NoSO_2_-Spont wine showed an intermediate level (2.3 g/L). In parallel, the non-sulphited variants exhibited a clear increase in lactic acid (0.21–0.27 g/L) relative to the must and the SO_2_Sc wine (0.07–0.12 g/L). This pattern should not be interpreted as direct evidence of malolactic fermentation, because it was not accompanied by a corresponding decrease in malic acid. Instead, it indicates differences in acid balance among the variants under low-SO_2_ conditions during the late stages of vinification [34]. Moreover, recent evidence indicates that SO_2_ management (timing and dose) influences the survival and activity of wine microbiota during ageing, with implications for acid composition and microbiological stability [36]. Citric acid remained at a low level (0.1–0.2 g/L; no significant differences) across the analysed variants, indicating that this fraction was not markedly affected under the conditions applied in the present study [37,38].

Succinic acid increased in all wines compared with the must (0.2 g/L), reaching a maximum in the spontaneous variant (NoSO_2_-Spont; 1.2 g/L) and intermediate values in the inoculated variants (NoSO_2_Sc and SO_2_Sc; 0.6–0.8 g/L). This increase is consistent with succinic acid being a typical by-product of yeast metabolism during alcoholic fermentation, the concentration of which may rise under higher metabolic load, variable microbiota, and nutrient limitations. In this context, the highest concentration observed under spontaneous fermentation can be interpreted as reflecting more complex microbial interactions and stress-related conditions characteristic of unmanaged fermentations [26].

YAN parameters confirmed substantial nitrogen consumption during fermentation, decreasing from 250 mg N/L in the must to 12–23 mg N/L in the wines. The lowest YAN was recorded in the spontaneous wine (NoSO_2_-Spont; 12 mg N/L), whereas residual values in the inoculated variants (NoSO_2_Sc and SO_2_Sc) were slightly higher (18–23 mg N/L). From an applied oenology perspective, low YAN in combination with residual sugars may be interpreted in two ways: on the one hand, it limits the potential for further growth of yeasts requiring high nitrogen availability; on the other hand, under low-SO_2_ conditions and higher pH, it does not eliminate the risk of activity by microorganisms better adapted to nutrient-poor wine environments, particularly during storage. Contemporary reviews emphasise that stabilising wines produced with reduced SO_2_ requires a hurdle approach, combining control of residual sugars, strict process hygiene, microbiological stabilisation, and careful management of ageing conditions [27].

From the standpoint of finished product stability, two factors are particularly important in combination: (I) higher residual sugars in the spontaneous wine (NoSO_2_-Spont) and (II) low SO_2_ levels in the non-sulphited variants. Such a combination may increase susceptibility to unwanted microbial activity and the risk of refermentation after bottling, especially under fluctuating storage temperatures. Accordingly, it is appropriate to relate the observed differences to the chosen fermentation strategy (NoSO_2_-Spont versus NoSO_2_Sc and SO_2_Sc) and SO_2_ management as key tools shaping process predictability and the microbiological shelf stability of the wines [27,36].

The increase in L* after fermentation (from 69.40 to 76.49–78.55) indicates a lighter colour relative to the must, which is typical of white wines owing to suspended particle sedimentation, colloidal changes, and the partial removal of colour-active compounds during fermentation and early stabilisation [31,39]. At the same time, the decrease in b* across all wines (from 11.06 to 2.52–6.21) reflects a weakening of the yellow component, likely associated with adsorption of a part of the phenolic fraction onto yeast lees and with lower intensity of phenolic oxidation and enzymatic/non-enzymatic browning than in the must [39,40]. The shift in a* values from slightly positive in the must (0.25) to negative in all wines (−0.57 to −2.26) further indicates a move towards greener and visually fresher colour tones. The most negative a* value observed in the NoSO_2_-Spont variant suggests the greatest shift towards greenish tones, whereas the SO_2_Sc and NoSO_2_Sc variants showed less pronounced changes.

Among the analysed variants, SO_2_Sc was the lightest wine and showed the lowest b* value, consistent with the protective effect of SO_2_ against oxidation and the formation of yellowing phenolic oxidation products, thereby promoting clarity and limiting colour deepening [31,40,41,42]. By contrast, NoSO_2_-Spont showed the highest b* and the most negative a* values, suggesting greater colour shift under conditions of higher microbiological variability and less controlled oxygen balance [39,40,43]. The NoSO_2_Sc variant showed intermediate colour parameters, indicating that inoculation may partly stabilise fermentation progress and mitigate unfavourable colour changes associated with oxygen exposure, even in the absence of SO_2_ [31,39,44]. Overall, these results highlight the importance of SO_2_ in limiting oxidative colour changes, while also indicating that an appropriate fermentation strategy, including inoculation, can support colour control in low-SO_2_ winemaking approaches [45].

A similar sensitivity of ‘Solaris’ to technological interventions has previously been reported. Zhang et al. [20] showed that different pre-fermentation treatments, including direct pressing, whole-cluster pressing, cold maceration, and skin fermentation, significantly affected the volatile composition, phenolic profile, and sensory expression of ‘Solaris’ wines. Although the present study focuses on SO_2_ management and yeast inoculation rather than juice-extraction strategy, the observed differences in acid balance, basic compositional parameters, and colour-related traits likewise support the view that ‘Solaris’ is highly responsive to technological decisions applied before and during fermentation.

### 2.3. Major Fermentation By-Products

Table 3 summarises the effect of fermentation management on key fermentation by-products in the finished wines produced under three variants: SO_2_Sc, NoSO_2_-Spont, and NoSO_2_Sc. All wines were produced from the same must and fermented under comparable temperature conditions (12 ± 0.5 °C), but they differ in microbial control strategy. Fermentation comprised approximately 10 days of vigorous alcoholic fermentation followed by around 2 months of slower fermentation. Subsequently, wines were racked off the lees and allowed to sediment naturally for additional months at 12 °C prior to bottling. This extended pre-bottling period is technologically relevant because many aroma-active compounds arise during fermentation and are particularly influential in young wines, while slow post-fermentation processes may stabilise or accentuate compositional differences [46].

Ethanol content was significantly lower in the NoSO_2_-Spont (12.9% *v*/*v*) than in the inoculated variants (NoSO_2_Sc and SO_2_Sc; 13.4–13.5% *v*/*v*) (Table 3). When interpreted alongside Table 1, this is consistent with significantly higher residual sugars in the NoSO_2_-Spont (glucose 2.4 g/L; fructose 4.5 g/L) compared with the inoculated wines (NoSO_2_Sc and SO_2_Sc; glucose 0.3–0.8 g/L; fructose 1.6–2.3 g/L). This pattern indicates less complete sugar conversion under spontaneous fermentation, which is frequently associated with variable microbial succession and late-fermentation constraints linked to ethanol stress and nutrient availability [26,47,48].

Glycerol was the most abundant by-product after ethanol. The highest glycerol concentration occurred in the NoSO_2_-Spont (14.5 g/L), followed by the NoSO_2_Sc (10.2 g/L), whereas the SO_2_Sc showed the lowest level (7.7 g/L). Glycerol formation depends on multiple factors, including yeast strain, sugar concentration, must pH, SO_2_ regime, and temperature, and it is commonly linked to yeast redox balancing and stress responses during fermentation [49]. Accordingly, the elevated glycerol in the NoSO_2_-Spont variant plausibly reflects a fermentation milieu characterised by broader microbial diversity and altered metabolic regulation relative to the more controlled SO_2_Sc fermentation, while early inoculation in the NoSO_2_Sc variant may have partially constrained microbial succession and moderated stress-associated glycerol accumulation compared with the fully spontaneous regime [26].

Higher alcohols constitute a major fraction of fermentation-derived volatiles. At moderate concentrations, they may contribute positively to aroma complexity, whereas at excessive levels they can produce pungent or solvent-like notes [50,51]. In the present study, the NoSO_2_-Spont variant exhibited the highest concentrations of isoamyl alcohol (115.9 mg/L), n-propanol (26.6 mg/L), and the sum of 2- and 3-methylbutanol (62.1 mg/L), while the SO_2_Sc variant showed the lowest levels for these compounds (57.0, 19.8, and 36.4 mg/L, respectively). Such increases are consistent with enhanced amino-acid catabolism and differences in nitrogen metabolism under spontaneous fermentation [26,52]. Notably, isobutanol followed the opposite trend, being highest in the SO_2_Sc variant (24.2 mg/L) and lowest in the NoSO_2_-Spont variant (15.0 mg/L), highlighting that individual higher alcohols may respond differently to the fermentation regime and nutrient status rather than changing uniformly [53].

Acetaldehyde is a key intermediate in yeast metabolism and an important component of wine redox chemistry; in young wines it is commonly reported at concentrations on the order of tens of mg/L and is also involved in pathways linked to ester formation [54,55]. In the present study, acetaldehyde was highest in the NoSO_2_-Spont variant (55.7 mg/L), followed by the NoSO_2_Sc variant (39.2 mg/L), whereas the SO_2_Sc variant showed the lowest concentration (34.9 mg/L). The higher acetaldehyde concentrations in the non-sulphited variants are consistent with reduced antimicrobial protection and potentially broader microbial activity during the extended pre-bottling period [36].

Acetoin, which may contribute buttery notes, can arise via citrate-related metabolism associated with lactic acid bacteria [56]. Here, acetoin was highest in the NoSO_2_-Spont variant (0.53 mg/L) and lowest in the NoSO_2_Sc variant (0.15 mg/L). This aligns with Table 1, where the non-sulphited wines exhibit higher lactic acid, suggesting greater activity of lactic acid bacteria and related acid biotransformations under low-SO_2_ conditions [34,36]. More broadly, non-*Saccharomyces* yeasts and mixed microbial communities can modulate carbonyl and ester formation, thereby shifting the overall aroma balance [26,57].

Acetate esters contribute to fruity aroma notes; isoamyl acetate (banana-like) and isobutyl acetate (tropical-fruit-like) are among the commonly discussed contributors [50]. In this study, isoamyl acetate and isobutyl acetate were highest in the NoSO_2_-Spont variant (0.23 and 0.29 mg/L, respectively), indicating enhanced formation of selected aroma-positive acetate esters under spontaneous conditions. By contrast, ethyl acetate was highest in the SO_2_Sc variant (44.8 mg/L) and lowest in the NoSO_2_-Spont variant (20.6 mg/L). This indicates that fermentation management shifted the balance among ester-related pathways rather than producing a uniform increase in all esters in the spontaneous variants. Importantly, ethyl acetate remained within ranges generally considered non-fault-forming in dry wines, where low to moderate concentrations may contribute fruity nuances, whereas very high levels are associated with solvent-like off-odours [50,58].

Volatile acidity (VA) was significantly higher in wines produced under non-sulphited conditions (0.78 g/L in NoSO_2_-Spont; 0.74 g/L in NoSO_2_Sc) than in SO_2_Sc (0.21 g/L). From a stability perspective, this is a key outcome. When interpreted together with Table 1, elevated VA in the non-sulphited variants is consistent with very low SO_2_ (total 3 mg/L) and, in NoSO_2_-Spont, higher residual sugars, which together may favour unwanted microbial activity during prolonged pre-bottling handling and storage [34,36,59]. Conversely, sulphiting combined with starter inoculation provides a more controlled microbial environment and coincided with the lowest VA, supporting the established role of SO_2_ management as a central tool for limiting spoilage development during ageing and storage [27,36].

Overall, Table 2 and Table 3 consistently indicate that NoSO_2_-Spont, followed by extended cool ageing and sedimentation prior to bottling, resulted in (I) less complete sugar conversion and lower ethanol, (II) higher glycerol and elevated concentrations of several higher alcohols, (III) increased acetaldehyde and acetoin, and (IV) substantially higher volatile acidity. In contrast, SO_2_Sc exhibited a more controlled compositional profile—particularly with respect to volatile acidity and carbonyl compounds—highlighting that microbial management is critical not only during alcoholic fermentation but also throughout prolonged pre-bottling storage at low temperatures [27,34,36]. These findings are in line with earlier observations that ‘Solaris’ wines may show substantial chemical and sensory variability depending on production conditions [19]. Our results extend this cultivar-specific evidence by showing that, even when the same must is used, differences in SO_2_ management and yeast inoculation are sufficient to markedly alter fermentation completeness, volatile acidity, and the profile of major fermentation by-products. A comparable effect of fermentation strategy has also been demonstrated in other white wine systems, where spontaneous vinification generated distinct microbiota, volatilome, and sensory attributes compared with inoculated fermentations, confirming the central role of microbiological control in shaping final wine composition [25].

### 2.4. Polyphenolic Compounds

Overall, the polyphenolic profile differed clearly among the three winemaking variants (Table 4). The lowest total polyphenol content was observed in SO_2_Sc (16.92 µg/mL), intermediate values in NoSO_2_-Spont (21.22 µg/mL), and the highest in NoSO_2_Sc (31.28 µg/mL), indicating that the non-sulphited inoculated variant shows the greatest overall retention of polyphenolic compounds. When the major phenolic groups are considered separately, phenolic acids and flavonols follow the same general ranking, with the lowest levels in SO_2_Sc and the highest in NoSO_2_Sc, while the spontaneous wine generally occupies an intermediate position. Flavan-3-ols increased in all wine variants relative to the must and represents the quantitatively dominant phenolic fraction, particularly in the non-sulphited wines. Stilbenes showed the least pronounced differentiation, although their total content was also highest in NoSO_2_Sc. Taken together, these results show that the three fermentation strategies differ not only in total polyphenol content, but also in the relative distribution of the main polyphenolic fractions.

Phenolic acids: among phenolic acids, the total content was lowest in SO_2_Sc (3.58 µg/mL), intermediate in NoSO_2_-Spont (5.23 µg/mL), and highest in NoSO_2_Sc (10.53 µg/mL), compared with 5.98 µg/mL in the must. This differentiation is driven mainly by caftaric acid, which showed the clearest variation among variants (1.00 µg/mL in SO_2_Sc, 1.83 µg/mL in NoSO_2_-Spont, and 6.32 µg/mL in NoSO_2_Sc), and by *p*-coumaric acid, which followed the same directional trend. The marked decrease in caftaric acid in SO_2_Sc is consistent with more efficient clarification and sedimentation after SO_2_ addition, which may favour the removal or immobilisation of hydroxycinnamates together with suspended solids and lees; part of this fraction may also be involved in oxidation-related transformations and partitioning phenomena during early wine stabilisation [60,61,62]. By contrast, the substantially higher caftaric acid level in NoSO_2_Sc may reflect greater retention of this fraction in the liquid phase and/or more limited oxidative losses during fermentation and lees contact under these conditions [63]. This interpretation is consistent with previous observations showing that phenolic composition and colour-related changes in white grape matrices may be shaped by oxidation and partitioning processes during processing rather than by ethanol-driven extraction alone [18,64]. A similar trend was observed for *p*-coumaric acid, which was lowest in SO_2_Sc and highest in NoSO_2_Sc, while gallic acid also increased in the non-sulphited variants. This is technologically relevant because hydroxycinnamates are not only contributors to the antioxidant potential of white wines, but also important substrates in oxidation pathways and, in the case of *p*-coumaric acid, potential precursors of microbiologically mediated transformations such as volatile phenol formation under less controlled conditions [40,65,66]. By contrast, coumaric acid and ferulic acid remained relatively stable across variants, suggesting lower sensitivity of these compounds to the applied fermentation strategies. From a white wine quality perspective, the higher total phenolic acid pool in NoSO_2_Sc may indicate greater antioxidant potential, but also a more reactive matrix, potentially more prone to browning and loss of aromatic freshness if oxygen exposure is not carefully controlled [40,61]. Thus, the phenolic acid profile differentiates the three winemaking strategies not only quantitatively, but also in terms of probable oxidative behaviour and technological stability during further ageing [17,67].

Flavonols showed the same overall ranking as phenolic acids, with the lowest total level in SO_2_Sc (0.15 µg/mL), intermediate values in NoSO_2_-Spont (1.32 µg/mL), and the highest in NoSO_2_Sc (2.20 µg/mL), compared with 0.45 µg/mL in the must. The strongest differentiation was observed for quercetin derivatives, especially quercetin 3-*O*-rhamnoside, quercetin 3-*O*-glucoside, and quercetin 3-*O*-rutinoside, all of which increased markedly in the non-sulphited wines. In SO_2_Sc, the low total flavonol content is consistent with more efficient clarification and sedimentation after SO_2_ addition, together with adsorption of flavonol glycosides onto yeast lees, which may reduce their measurable concentration in young white wines [68,69]. By contrast, the substantially higher levels observed in NoSO_2_-Spont and particularly in NoSO_2_Sc may reflect greater retention of fine skin-derived and colloidal material in the liquid phase during fermentation, as well as less intensive removal of this fraction during clarification and lees separation [70,71,72]. This interpretation is supported by the pronounced increase in quercetin 3-*O*-rhamnoside (0.05 µg/mL in SO_2_Sc, 0.70 µg/mL in NoSO_2_-Spont, and 1.19 µg/mL in NoSO_2_Sc), quercetin 3-*O*-glucoside (0.03, 0.38, and 0.55 µg/mL, respectively), and quercetin 3-*O*-rutinoside (0.01, 0.15, and 0.25 µg/mL, respectively), whereas myricetin 3-*O*-glucoside and dihydroquercetin 3-*O*-rhamnoside remained comparatively stable across variants. From a white wine quality perspective, increased flavonol content may enhance antioxidant potential and contribute to phenolic complexity, but it may also increase matrix reactivity because flavonols can participate in coupling reactions with oxidation products of hydroxycinnamates, thereby promoting browning and loss of aromatic freshness under suboptimal oxygen management [73,74,75]. Thus, similarly to phenolic acids, the highest flavonol pool in NoSO_2_Sc may indicate greater phenolic retention, but at the same time a greater need for strict oxygen control and carefully selected antioxidant protection during ageing and storage [40,71].

Flavan-3-ols increased in all wine variants relative to the must and represented the quantitatively dominant phenolic fraction. Their total content rose from 7.31 µg/mL in the must to 11.96 µg/mL in SO_2_Sc, 13.31 µg/mL in NoSO_2_-Spont, and 16.96 µg/mL in NoSO_2_Sc. The most pronounced increases were observed for (−)-epicatechin, epicatechin gallate, and both procyanidin type A and B fractions, indicating that fermentation favoured the retention and/or transfer of these compounds into the wine matrix. Because the wines were produced without skin maceration, these differences are more likely associated with the contribution of seed-derived phenolics and fine suspended material retained after pressing, as well as with differences in clarification efficiency and lees interactions, rather than with ethanol-driven extraction from skins [68,71]. The progressive increase from SO_2_Sc to NoSO_2_Sc suggests that non-sulphited conditions favoured greater preservation of this fraction, likely owing to reduced clarification efficiency and longer persistence of suspended solids and colloidal material during fermentation. From a technological and sensory point of view, this is important because flavan-3-ols are key contributors to bitterness, astringency, and structural perception, even in white wines, while at the same time increasing the oxidative reactivity of the phenolic matrix when present together with elevated hydroxycinnamate levels [71,73]. Therefore, the strong enrichment of flavan-3-ols in NoSO_2_Sc supports the interpretation that this variant retained the largest share of phenolic material, but also generated the most reactive phenolic matrix, with potentially greater susceptibility to browning and loss of freshness during ageing if oxygen exposure is not tightly controlled.

Stilbenes: among stilbenes, the total content was 1.23 µg/mL in SO_2_Sc, 1.36 µg/mL in NoSO_2_-Spont, and 1.59 µg/mL in NoSO_2_Sc, compared with 0.91 µg/mL in the must. The clearest changes concerned cis-resveratrol and cis-piceid, both of which increased in the non-sulphited variants, particularly in NoSO_2_Sc. Trans-resveratrol also increased in NoSO_2_Sc, whereas trans-piceid showed the highest value in SO_2_Sc (0.67 µg/mL) and intermediate levels in both non-sulphited wines (0.60 µg/mL). Although stilbenes occur in white wines at relatively low concentrations and are not considered major direct determinants of sensory profile, they are important as markers of phenolic retention, bioactive potential, and redox-related evolution during vinification and ageing [76,77]. The differences observed among variants may reflect variant-dependent preservation and isomerisation pathways operating during fermentation and lees contact. In particular, the increase in the cis forms suggests that non-sulphited conditions may have favoured structural transformations within the stilbene fraction and/or differential preservation of these compounds during wine development. This interpretation is consistent with evidence that trans-resveratrol may undergo isomerisation to cis-resveratrol and further transformation reactions under appropriate environmental conditions, including light-induced processes [78,79,80]. From a practical perspective, the stilbene profile supports the interpretation that reduced-SO_2_ strategies not only affected the bulk phenolic composition, but also modified the behaviour of minor bioactive compounds that may serve as sensitive indicators of wine-processing conditions and matrix evolution.

### 2.5. Sensory and Technological Implications

Flavan-3-ols contribute to perceived body, bitterness, and astringency [81,82]. In white wines, they may therefore strengthen structure; however, when combined with a high hydroxycinnamate pool, they also increase the oxidative reactivity of the matrix. Monomeric and oligomeric flavan-3-ols can condense with oxidation products (quinones), supporting reactions that lead to browning and loss of aromatic freshness [40,73]. Consequently, non-sulphited variants require more rigorous oxygen control and deliberate management of turbidity and clarification in order to balance desirable structure with preservation of freshness and colour [68].

**Figure 1:**
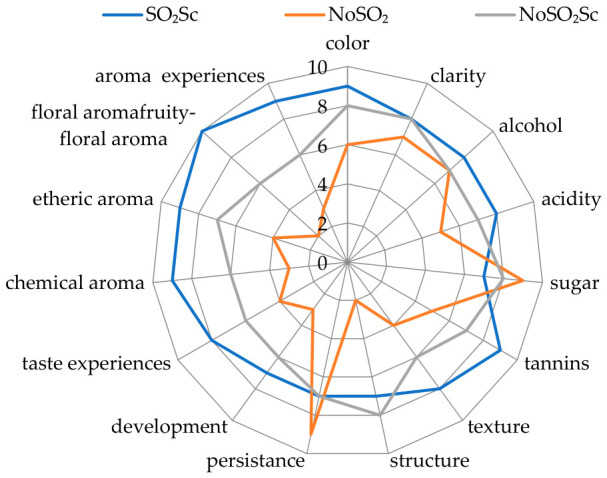
Sensory evaluation results for wines produced under three fermentation variants (SO_2_Sc, NoSO_2_-Spont, NoSO_2_Sc—see explanation under Table 1).

In descriptive sensory terms, the wines differed clearly across the evaluated attributes (Figure 1). The SO_2_Sc wine showed the most favourable overall sensory profile, receiving the highest mean scores for colour, acidity, tannins, texture, development, taste experiences, chemical aroma, etheric aroma, floral/fruity-floral aroma, and overall aroma experiences, while also maintaining high ratings for clarity and alcohol perception. The NoSO_2_Sc wine displayed an intermediate sensory profile, with values generally between those of SO_2_Sc and NoSO_2_-Spont, and was distinguished in particular by high clarity and the highest score for structure. By contrast, the NoSO_2_-Spont wine was characterised by comparatively high scores for sweetness and persistence, but had lower ratings for acidity, tannins, texture, structure, development, taste experiences, and aroma-related attributes. Taken together, these results indicate that the spontaneous non-sulphited variant was perceived as sweeter and more persistent, but also less structured, less developed, and less aromatically expressive than the inoculated wines. Overall, the sensory data support the conclusion that the sulphited inoculated variant provided the most favourable and broadly accepted sensory profile under the conditions of the present study.

Because all variants were prepared from the same ‘Solaris’ must (single press run; destemming, crushing, and pressing for ~2 h to 2.5 bar) and fermented under identical conditions, the observed differences in stilbene content and profile are unlikely to reflect differential ethanolic extraction from skins (which did not occur in this design). Instead, they should be attributed to processes operating after the must was split into variants, including redox stability, interactions with lees/sediment, and enzymatic–chemical transformations during fermentation and ageing on lees [76,77]. In this context, stilbenes (resveratrol and its glucosides, i.e., piceid) act as sensitive indicators of the balance between retention in the liquid phase and loss/transformation during processing.

Total stilbenes increased progressively across variants (0.91 µg/mL in the must > 1.23 µg/mL in SO_2_Sc > 1.36 µg/mL in NoSO_2_-Spont > 1.59 µg/mL in NoSO_2_Sc), with the most pronounced increase observed in the NoSO_2_Sc variant. In this variant, both trans-resveratrol (0.03 µg/mL in the must > 0.08 µg/mL in NoSO_2_Sc) and cis-resveratrol (0.18 µg/mL in the must > 0.35 µg/mL in NoSO_2_Sc) increased alongside a relatively high pool of glucosides (piceid). This pattern suggests the co-occurrence of two mechanisms: (I) partial conversion of glucosides to the aglycone, driven by microbial β-glucosidase activity, which may be more apparent in microbiologically complex systems (NoSO_2_-Spont and/or NoSO_2_Sc) [83]; and (II) trans/cis isomerisation influenced by environmental conditions and secondary reactions (including light- and oxidation-related processes), consistent with resveratrol being prone to structural transformation [80]. Notably, the increase in the cis fraction (especially cis-resveratrol and cis-piceid) in the non-sulphited variants is consistent with the concept that reduced antioxidant protection and altered secondary-reaction dynamics can shift the isomeric equilibrium even in the absence of additional skin-derived extraction.

In white wines, stilbene concentrations are typically too low to exert a direct impact on the sensory profile; however, they are relevant as markers of bioactivity (resveratrol > piceid) and as indicators of redox evolution and wine–lees interactions. Within low-SO_2_ strategies, SO_2_ reduction necessitates more rigorous control of oxygen exposure and clarification, because the same conditions that favour higher phenolic loads may simultaneously increase the reactivity of hydroxycinnamates and flavan-derived fractions associated with browning and loss of aromatic freshness [40,68]. At the same time, interest is growing in SO_2_-limiting approaches that incorporate stilbene-rich resources (e.g., vine-shoot/cane extracts) as potential protective components in wines produced with reduced sulphiting [84].

In sensory terms, the present ranking of the wines (SO_2_Sc > NoSO_2_Sc > NoSO_2_-Spont) is consistent with the broader literature on controlled versus spontaneous fermentations. Wines inoculated with *S. cerevisiae* are frequently described as showing cleaner, more controlled, and more favourably expressed sensory profiles, often with clearer fruity and floral character and better overall aromatic definition, as also highlighted in reviews on the role of wine yeasts in aroma formation and in recent studies comparing commercial yeast strains in wine production [85,86]. By contrast, spontaneous fermentations may generate greater complexity, but they are also more often associated with higher sensory variability and, depending on microbial succession and process control, with less fresh, less clean, and less harmoniously expressed aroma profiles. In such wines, the sensory picture may shift towards weaker aromatic precision and occasionally more herbaceous, nutty, solvent-like, or otherwise less expressive notes, particularly when volatile acidity and other spoilage-associated metabolites increase; this pattern is consistent with the findings of Bekris et al. [25], who report distinct sensory attributes in spontaneously vinified wines compared with inoculated fermentations, and with broader evidence linking spontaneous fermentation to a higher risk of volatile-acidity-related sensory deviation [87]. In this context, the weak nose, reduced freshness, and lower overall aromatic appeal observed in the NoSO_2_-Spont variant are in line with published reports showing that spontaneous vinification often yields less predictable sensory outcomes than *S. cerevisiae*-inoculated fermentations.

This sensory differentiation is also consistent with previous work on ‘Solaris’ wines from Sweden, where a broad sensory space and limited agreement on a single “typical” profile were reported for this cultivar [21]. More recent work by Toldam-Andersen et al. [88] further shows that SO_2_ management during vinification significantly influences flavour development and sensory properties during storage, confirming the importance of sulphite strategy for the final sensory profile of wines from this cultivar. The present results refine these observations by showing that, even within a single must source, differences in SO_2_ management and yeast inoculation are sufficient to produce either more controlled and favourably perceived sensory profiles or, conversely, less predictable and aromatically weaker outcomes.

When the chemical and sensory results are interpreted jointly, the three winemaking strategies differed markedly in the overall quality of the final wines. Under the conditions of the present study, SO_2_Sc may be regarded as the most favourable variant, combining the best sensory performance with the most controlled chemical profile, including the lowest volatile acidity, low residual sugars, and the brightest colour [88]. In contrast, NoSO_2_-Spont showed the least favourable overall profile, with weaker sensory performance—especially in aroma freshness and harmony—together with less complete fermentation and higher volatile acidity, consistent with the lower predictability of spontaneous fermentations and their greater susceptibility to quality deviations [25,89]. The NoSO_2_Sc wine occupies an intermediate position, showing advantages in phenolic retention and sensory perception over the spontaneous wine, but not matching the chemical stability and balance of SO_2_Sc [89]. These results suggest that, within the studied technological conditions, the combined use of sulphiting and inoculation provided the most reliable route to a chemically stable and sensorially favourable white wine, whereas spontaneous fermentation without SO_2_ was associated with a greater risk of quality deterioration [25,89].

### 2.6. Principal Component Analysis

**Figure 2:**
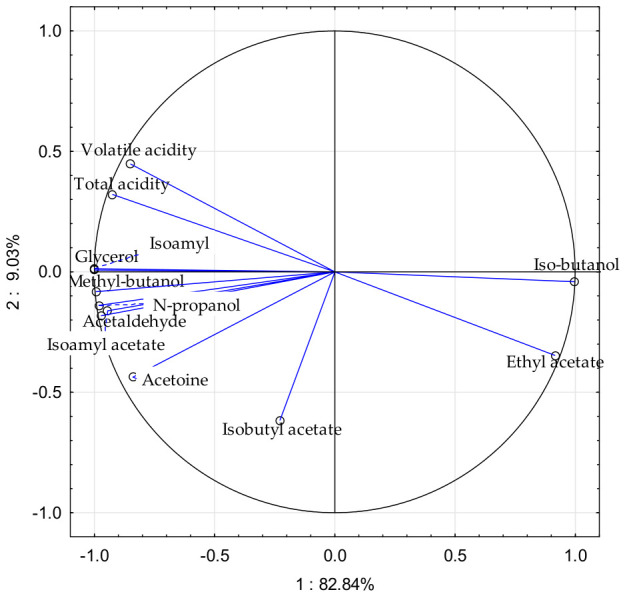
PCA loading plot (correlation circle) of major fermentation by-products in wines produced under three fermentation variants (NoSO_2_-Spont, NoSO_2_Sc, SO_2_Sc; see explanation under Table 1). PC1 explains 82.84% and PC2 9.03% of the total variance. Vector direction indicates the association with principal components, vector length reflects the strength of representation, and angles between vectors indicate correlations among variables.

The Principal Component Analysis (PCA) loading plot summarises the relationships among the measured fermentation by-products and related parameters in the analysed wines (Figure 2). PC1 explains 82.84% of the total variance, while PC2 accounts for 9.03%; together, they capture 91.87% of the variability, indicating that the two-dimensional representation provides a comprehensive overview of the main compositional gradients.

Along PC1, the strongest positive contributions are observed for isobutanol and ethyl acetate, whose vectors extend furthest towards the positive x-axis. By contrast, volatile acidity, total acidity, glycerol, isoamyl alcohol, n-propanol, methyl-butanol, and acetaldehyde are positioned on the negative side of PC1, indicating an opposite association pattern relative to isobutanol and ethyl acetate. This configuration suggests that PC1 primarily reflects a contrast between isobutanol/ethyl acetate and a group of acidity- and fermentation-related variables.

PC2 further differentiates variables associated with acidity from those located in the lower part of the plot. Volatile acidity and total acidity show positive loadings on PC2, whereas ethyl acetate, isobutyl acetate, acetoin, and isoamyl acetate are oriented towards negative PC2 values. The proximity and similar direction of vectors indicate positive correlations, as observed for volatile acidity and total acidity, whereas vectors oriented in opposite directions suggest negative associations, for example between acidity-related parameters and ethyl acetate.

Variables with longer vectors, particularly isobutanol, ethyl acetate, volatile acidity, and total acidity, contributed more strongly to the structure described by PC1 and PC2, whereas shorter vectors indicate weaker representation in the first two principal components.

Overall, the PCA plot indicates that variability in the dataset is driven mainly by the balance between ester- and alcohol-related variables and acidity-related parameters. As the figure presents variable loadings without sample scores, the PCA should be interpreted as an exploratory tool describing relationships among variables rather than direct separation between fermentation variants.

## 3. Materials and Methods

### 3.1. Characteristics of the Area of Research and Plant Material

Grapes were harvested from the commercial Pałac Rajkowo Vineyard in Smolęcin, near Szczecin (north-western Poland). Most of the West Pomeranian Province falls within zone 7A on Heinz and Schreiber’s map of plant frost-resistance zones. However, in the Szczecin area and the adjacent northern region, minimum temperatures typically range from −12 °C to −15 °C, which corresponds to conditions characteristic of zone 7B. The average temperature during the growing season (April–October) between 1951 and 2021 was 13.7 °C and rainfall was 391 mm [90]. Vineyard soil was an agricultural soil with a natural profile, developed from silt loam, with pH 6.9, relatively high water-holding capacity, and optimal mineral content [91].

The vines were grafted onto SO_4_ rootstock and planted in 2016 with a north–south row orientation at a spacing of 1.0 m × 2.3 m. Vines were trained to a single Guyot system and vertically positioned with eight shoots; each shoot carried two clusters. Standard vineyard management practices, including pest and disease control, were applied during both growing seasons.

### 3.2. Variety Description and Wine Production

The experiment was conducted using white-skinned grapes of *Vitis vinifera* L. cv. ‘Solaris’, a German-bred cultivar increasingly cultivated in cool-climate regions. Grapes were harvested at technological maturity (23.7 °Brix; pH 3.3 ± 0.1) and processed immediately after picking.

Must preparation and homogenisation

Whole clusters were destemmed and crushed, and must was obtained using a pneumatic press operated up to 2.5 bar for 2 h. The entire press-run must (combined fractions from the complete pressing) was collected as a single batch, thoroughly mixed (homogenised), and then divided into fermentation variants to ensure identical starting material. The must was clarified by cold settling for 12 h at 10 °C and racked off the gross lees prior to fermentation.

Experimental design (variants and replication)

Three vinification variants were prepared in triplicate in 50 L stainless steel tanks:

1. NoSO_2_-Spont: spontaneous fermentation without SO_2_ addition and without inoculation (indigenous microbiota).
2. NoSO_2_Sc: no SO_2_ addition; inoculated with *Saccharomyces cerevisiae* ES181.
3. SO_2_Sc: sulphited must; inoculated with *S. cerevisiae* ES181.

Sulphiting procedure (SO_2_Sc only)

In SO_2_Sc, potassium metabisulphite (K_2_S_2_O_5_; E224; ≥56% SO_2_) was added at 0.8 g/10 L, corresponding to 45 mg/L total SO_2_. Metabisulphite was dissolved in a small volume of must (or 20–50 mL of water), added to the tank, and mixed thoroughly. The must was allowed to equilibrate briefly (typically 30–60 min) before inoculation.

Yeast inoculation (NoSO_2_Sc and SO_2_Sc)

A commercial active dry yeast, *Saccharomyces cerevisiae* ES181 (Enartis S.r.l., San Martino Trecate, Italy; distributed by BS vinárske potreby s.r.o., Pezinok, Slovakia), was applied at 30 g/hL. Yeast was rehydrated in 150 mL of water at 35 °C and added to the must according to the assigned variant.

The inoculum was added according to the manufacturer’s instructions. For inoculated variants, the target inoculum level was standardised, whereas actual yeast counts measured in musts before fermentation are reported in Table 1. Before fermentation values refer to must sampled after clarification and after variant-specific SO_2_/inoculation treatment, immediately before the onset of active fermentation.

Nitrogen management (YAN correction)

Must YAN was 137 mg N/L and was adjusted to 250 mg N/L using diammonium phosphate (DAP), i.e., a correction of +113 mg N/L. The calculated DAP dose was 0.54 g/L (equivalent to 54 g/hL; 5.4 g per 10 L) and was applied in two split additions: (I) 10–15 g/hL at fermentation onset (12–24 h after inoculation, once active fermentation was evident), and (II) 10–15 g/hL at approximately one-third sugar depletion (e.g., from ~24 °Brix to ~16–17 °Brix).

Fermentation conditions, reductive handling, and post-fermentation operations

Fermentation was conducted at 12 ± 0.5 °C under reductive conditions (sealed tank lids and fermentation airlocks). No forced aeration or oxygenation was applied during fermentation or post-fermentation handling. The process comprised approximately 10 days of vigorous alcoholic fermentation, followed by about 2 months of slow fermentation. For Table 1, the term “before” refers to the sampling point on the day of experiment establishment, immediately after preparation of each fermentation variant and before sealing the fermentation vessels. In the inoculated variants (NoSO_2_Sc and SO_2_Sc), samples were collected immediately after addition of the rehydrated yeast suspension to the must. In the spontaneous variant (NoSO_2_-Spont), samples were collected after transfer of the must to the fermentation tank. Thus, the “before” values represent the viable yeast population present in each variant at time 0, i.e., immediately prior to the onset of active fermentation under the assigned treatment conditions. The initial differences in yeast counts among variants therefore reflect the experimental design, including inoculation and SO_2_ addition. After completion, wines were racked off the lees and left to sediment naturally for additional months at 12 °C prior to bottling. The term “after” refers to the sampling point after completion of alcoholic fermentation and after the same post-fermentation storage and natural sedimentation period applied to all variants, immediately before bottling. At this stage, samples were collected for yeast enumeration and for the determination of the remaining analytical parameters. All variants were bottled on the same date, and analyses were performed after the same post-fermentation storage period.

SO_2_ analysis

Free and total SO_2_ were determined by the aeration–oxidation method according to OIV-MA-AS323-04A1 [92] and OIV-MA-AS323-04A2 [93].

### 3.3. Yeast, Identification and Content

To determine viable yeast counts in musts and wines, yeast enumeration was performed by the surface spread plate method on a YPG medium (Yeast Extract–Peptone–Glycerol Medium; Sigma-Aldrich, Merck Life Science Sp. z o.o., Poznań, Poland), following Błaszak et al. [2]. Musts and wine samples were subjected to serial ten-fold decimal dilutions in a sterile diluent. Aliquots of 0.1 mL of appropriate dilutions were spread evenly on the surface of YPG agar plates and incubated for 3 days at 25 °C. After incubation, colonies were counted using an eCount Colony Counter (Heathrow Scientific, Vernon Hills, IL, USA), and results were expressed as colony-forming units per millilitre (CFU/mL) of wine. Analyses were performed in five replicates per sample/variant.

Sequence-based molecular identification was performed only for representative yeast isolates recovered from the NoSO_2_-Spont wine after fermentation, in order to characterise the surviving culturable yeast population in the spontaneous variant. This analysis was not carried out for isolates from the NoSO_2_Sc or SO_2_Sc variants. No analysis was carried out on these variants because it is known that *S. cerevisiae* ES181 was introduced there. Species identification of yeast grown on the medium was made using a standard sequencing technique. DNA region ITS (Internal transcribed spacer) (primers for ITS1: TTC GTA GGT GAA CCT GCG G; ITS4: TCC TCC GCT TAT TGA TAT GC). Ge-nomic DNA was isolated using a method based on the Genomic Mini AX Bacteria+ (A&A Biotechnology, Poland) with additional mechanical lysis of the sample in FastPrep24 using zirconium balls. The DNA fragments obtained from the amplification reaction were purified using the kit Clean-Up AX (A&A Biotechnology, Gdańsk, Poland). PCR products were placed in 10mM Tris-HCl pH 8.0 buffer, diluted to a concentration of 50 ng/μL and sequenced (Macrogen, Holland). The obtained sequences were analysed in CLCMain Workbench 8 and BLAS programme [94].

### 3.4. Compounds in Wine—Identification and Content

pH was measured potentiometrically according to the OIV method OIV-MA-AS313-15 [95] using an Elmetron laboratory pH metre after calibration with SI-traceable buffer solutions at 20 °C. Measurements were performed directly in wine samples at 20–25 °C, in duplicate, and results were expressed to two decimal places as mean values. Total acidity (TA) was determined according to the OIV method OIV-MA-AS313-01 [96] by potentiometric titration with standard NaOH to pH 7.0 using an Elmetron pH metre (Elmerton, Zabrze, Poland). Dissolved CO_2_ was removed prior to analysis, and results were expressed as g/L tartaric acid. Ethanol (% *v*/*v*) was determined according to the OIV method OIV-MA-AS312-01A [97] and expressed as alcoholic strength by volume at 20 °C.

Yeast assimilable nitrogen (YAN) was calculated as the sum of ammonium nitrogen and primary amino nitrogen (NOPA). Ammonium nitrogen was determined according to OIV-MA-AS322-01 [98], whereas NOPA was determined by the *o*-phthaldialdehyde spectrophotometric method according to Dukes and Butzke [99]. Results were expressed as mg N/L.

Glucose and fructose were determined using a WineScan analyser (FOSS, Hillerød, Denmark). The measurements were based on Fourier transform infrared (FTIR) spectroscopy with the manufacturer’s calibration for wine analysis. Samples were analysed according to the manufacturer’s operating procedure, and the results are expressed as g/L. Glycerol and organic acids (tartaric, malic, citric, lactic, and succinic acids) were determined by HPLC using a Shimadzu Nexera XR system (Shimadzu, Japan). Prior to analysis, wine samples were clarified by removal of suspended solids by centrifugation and/or membrane filtration. Glycerol was quantified using an Asahipak NH2P-50 column (250 × 4.6 mm; Shodex/Showa Denko Europe, Germany), whereas organic acids were separated chromatographically and quantified by external calibration with analytical standards. Results were expressed as g/L. Ethyl acetate, higher alcohols (propanol, isobutanol, and 2- and 3-methylbutanol), acetoin, isoamyl acetate, and isobutyl acetate were determined by direct-injection gas chromatography using a Shimadzu GC-2030 gas chromatograph (Shimadzu, Japan) equipped with a flame ionisation detector (FID), under routine laboratory conditions for volatile fermentation compounds and with quantification based on calibration with analytical standards. Acetaldehyde was measured spectrophotometrically according to Di Stefano and Ciolfi [100], whereas volatile acidity was determined according to the OIV reference method OIV-MA-AS313-02 [101] by steam distillation followed by titration with standard sodium hydroxide and expressed as g/L acetic acid.

### 3.5. Identification of Phenolic Compounds with the UPLC-PDA/MS Method

Polyphenolic compounds were analysed using a UPLC–PDA–MS/MS Waters ACQUITY system (Waters, Milford, MA, USA) equipped with a binary solvent manager, sample manager, column manager, PDA detector and a tandem quadrupole mass spectrometer (TQD) with electrospray ionisation (ESI). Separation was performed on a BEH C18 column (100 mm × 2.1 mm i.d., 1.7 µm; Waters) maintained at 50 °C. The mobile phases were A: 0.1% formic acid in water (*v*/*v*) and B: 0.1% formic acid in acetonitrile (*v*/*v*). The gradient programme was: 0 min, 5% B; 0–8 min, linear increase to 100% B; 8–9.5 min, column washing and return to the initial conditions. The flow rate was 0.35 mL/min, and the injection volume was 5 µL (partial loop with needle overfill).

TQD parameters were as follows: capillary voltage 3.5 kV; cone voltage 30 V (positive and negative ion modes); source temperature 250 °C; desolvation temperature 350 °C; cone gas flow 100 L/h; desolvation gas flow 800 L/h. Argon was used as collision gas at 0.3 mL/min. Polyphenolic detection and identification were based on PDA spectra, precursor m/z values and characteristic product ions obtained after collision-induced dissociation (CID). Quantification was performed in Multiple Reaction Monitoring (MRM) mode using compound-specific transitions; cone voltage and collision energy were optimised manually for each analyte, with a dwell time of at least 25 ms.

Prior to analysis, wine samples were filtered through a 0.45 µm membrane filter (Merck Millipore KGaA, Darmstadt, Germany) and injected directly. Quantification was achieved using external calibration with analytical standards for all analysed polyphenolic compounds, using solutions of known concentrations in the range 0.05–5 mg/mL with linearity R^2^ ≥ 0.9998. All determinations were performed in triplicate and expressed as mg/L. Data acquisition and processing were carried out using Waters MassLynx v.4.1. The analysed polyphenolic compounds, together with their retention and mass spectrometric identification parameters, are listed in Table 5.

### 3.6. Colour Measurement

Colour was expressed using the CIE L*a*b* colour space, where L* indicates lightness (L* = 0, black; L* = 100, white), a* represents the green (−a*) to red (+a*) axis, and b* represents the blue (−b*) to yellow (+b*) axis. Colour coordinates were measured under the D65 illuminant and the 10° standard observer using a Konica Minolta CM-700d spectrophotometer (Konica Minolta, Inc., Tokyo, Japan). Measurements were performed in a glass cuvette using 25 replicate readings per sample, and the mean values were used for statistical analysis.

### 3.7. Sensory Evaluation

Wines were subjected to sensory evaluation using a semi-trained consumer panel. The panel consisted of 35 assessors who were informed about the purpose of the evaluation and received a short introduction to the assessed attributes and the scoring system prior to the test. The assessors were not professional tasters. The panel included both male and female participants aged 22–60 years.

Wine samples (30 mL) were served at 10 °C in 100 mL wine glasses and evaluated under consistent conditions. The three wine variants assessed were: SO_2_Sc, NoSO_2_-Spont, and NoSO_2_Sc. Samples were coded using random three-digit numbers and presented to each assessor in a randomised order to minimise order and carry-over effects. Between samples, assessors rinsed their mouths with water and consumed plain baguette, with an approximately 2 min break between evaluations.

Each sample was rated on a structured 0–10 scale (0 = not perceived/very low; 10 = very intense/very high) for the following attributes: colour, clarity, alcohol perception, acidity, sweetness (sugar), tannins, texture, structure, persistence, development (overall perceived complexity/maturity), overall taste impression, overall aroma impression, and selected aroma descriptors (floral aroma, etheric aroma—solvent-like notes, and chemical aroma—chemical/off-note character). Each of the 35 assessors evaluated all three wine variants twice in two independent blind repetitions conducted under the same testing conditions. Although individual scores showed minor variation between repetitions, the general direction of scoring remained similar, indicating a consistent tendency of the same assessor to evaluate a given wine in a comparable way across both assessments. For each attribute, individual scores were averaged across assessors, and the resulting mean values were visualised as a radar chart (spider plot) to compare sensory profiles among wine variants. As no inferential statistical analysis was performed, sensory results were treated as descriptive/exploratory and used to support interpretation of chemical and technological differences among variants rather than to claim statistically confirmed differences.

### 3.8. Statistical Analysis

All statistical analyses were performed using Statistica 12.5 (StatSoft Polska, Kraków, Poland). Data were analysed by one-way analysis of variance (ANOVA). When significant effects were detected, mean values were compared using Tukey’s HSD post hoc test. Differences were considered significant at *p* < 0.05.

## 4. Conclusions

Fermentation strategy is the primary factor shaping differences in the chemical composition and sensory profile of the wines produced under the three variants—NoSO_2_-Spont, NoSO_2_Sc, and SO_2_Sc—despite identical must origin and comparable fermentation conditions. The SO_2_Sc variant provided the most controlled outcome and the highest mean sensory acceptance, characterised by the lowest volatile acidity and the most favourable colour profile (higher L, lower b), which together indicate greater compositional stability during the prolonged cool pre-bottling period. By contrast, NoSO_2_-Spont was associated with less complete fermentation (higher residual sugars and lower ethanol) and the highest volatile acidity, indicating increased susceptibility to microbiological instability and lower process predictability under non-sulphited conditions. The NoSO_2_Sc variant showed an intermediate profile and partially mitigated deviations relative to NoSO_2_-Spont; however, volatile acidity remained elevated, and viable yeast counts in the finished wine were the highest, underscoring the need for additional stabilisation measures prior to bottling. The behaviour of volatile acidity across the studied variants further confirms that SO_2_ management is a key determinant of technological stability, as the non-sulphited wines showed a less favourable profile in this respect, indicating a greater susceptibility to quality deterioration under reduced-SO_2_ conditions. Changes in fermentation-derived volatiles were compound-specific, reflecting pathway rebalancing rather than uniform shifts across whole classes of compounds, which indicates that aroma optimisation requires regime-specific process control rather than reliance on inoculation as a single intervention. Polyphenolic composition was also strongly influenced by SO_2_ management and solids/lees dynamics; the non-sulphited variants, particularly NoSO_2_Sc, showed a higher overall phenolic load, including phenolic acids, flavonols, flavan-3-ols, and stilbenes. Although the increased phenolic load observed in wines produced without SO_2_ may enhance intrinsic antioxidant potential, it may also increase matrix reactivity and susceptibility to oxidative change, including colour deepening and loss of aromatic freshness, when oxygen management is insufficient. Overall, the production of wines with reduced SO_2_ requires a hurdle approach combining stringent oxygen management, control of residual fermentable sugars, strict hygiene, deliberate clarification and lees handling, and, where appropriate, microbiological stabilisation to limit volatile acidity development and reduce the risk of post-bottling refermentation.

## Figures and Tables

**Table 1:** Yeast count before and after fermentation in three variants of the process.

Variants **	Before *	After
	log10 CFU/mL	
NoSO_2_-Spont	4.88 d ***	1.48 a
NoSO_2_Sc	5.51 f	4.40 c
SO_2_Sc	5.40 e	3.63 b

Explanation: * Sampling dates “before” and “after” fermentation are described in the methodology, Section 3.2; ** NoSO_2_-Spont: must containing native microorganisms and undergoing spontaneous fermentation without sulphurisation or inoculation with a commercial yeast strain; NoSO_2_Sc: must containing native microorganisms, inoculated with a commercial strain of *S. cerevisiae* without sulphurisation; SO_2_Sc: must subjected to sulphurisation and inoculated with a commercial strain of *S. cerevisiae*; *** Mean values followed by the same letter do not differ significantly at *p* < 0.05 according to Tukey’s HSD test.

**Table 2:** Basic parameters of must (reference) and wines produced under three fermentation variants: NoSO_2_-Spont, NoSO_2_Sc, SO_2_Sc (see explanation under Table 1).

Parameters		Must	SO_2_Sc **	NoSO_2_-Spont	NoSO_2_Sc
pH		3.27 a	3.38 ab	3.46 b	3.41 b
Total SO_2_ (mg/L)		5 a	44 b	6 a	7 a
Free SO_2_ (mg/L)		3 a	27 b	3 a	3 a
Glucose (g/L)		108 c	0.3 a	2.4 b	0.8 a
Fructose (g/L)		122 c	1.6 a	4.5 b	2.3 a
Titratable acidity, TA (g/L)		7.47 c	7.02 a	7.31 b	7.57 c
Tartaric acid (g/L)		4.5 c	4 b	3.5 a	3.8 ab
Malic acid (g/L)		2.5 c	2.1 a	2.3 b	2.5 c
Citric acid (g/L)		0.2 a	0.2 a	0.1 a	0.2 a
Lactic acid (g/L)		0.07 a	0.12 a	0.21 b	0.27 b
Succinic acid (g/L)		0.2 a	0.6 b	1.2 c	0.8 b
YAN (mg/L)		250 c	18 b	12 a	23 b
	L *	69.40 a	78.55 c	77.47 bc	76.49 b
Colour CIE	a *	0.25 a	−0.57 b	−2.26 d	−0.94 c
	b *	11.06 d	2.52 a	6.21 c	3.79 b

Explanation: * Mean values followed by the same letter are not significantly different at *p* < 0.05 (Tukey’s HSD test). ** see explanation under Table 1.

**Table 3:** Major fermentation by-products in wines produced under three fermentation variants.

Parameters	SO_2_Sc **	NoSO_2_-Spont	NoSO_2_Sc
Ethanol % *v*/*v*	13.5 b	12.9 a	13.4 b
Glycerol (g/L)	7.7 a	14.5 c	10.2 b
Iso-amyl (mg/L)	57.0 a	115.9 c	81.5 b
Iso-butanol (mg/L)	24.2 c	15.0 a	20.2 b
N-propanol (mg/L)	19.8 a	26.6 c	21.6 b
2 and 3-Methyl-butanol (mg/L)	36.4 a	62.1 c	44.7 b
Ethyl acetate (mg/L)	44.8 c	20.6 a	25.9 b
Acetoin (mg/L)	0.21 b	0.53 c	0.15 a
Acetaldehyde (mg/L)	34.9 a	55.7 c	39.2 b
Isoamyl acetate (mg/L)	0.12 a	0.23 b	0.14 a
Isobutyl acetate (mg/L)	0.19 a	0.29 c	0.24 b
Volatile acidity (g/L acetic acid)	0.21 a	0.78 b	0.74 b

Explanation: ** NoSO_2_-Spont, NoSO_2_Sc, SO_2_Sc (see explanation under Table 1).

**Table 4:** Polyphenolic compound content (µg/mL) in must (reference) and wines produced under three fermentation variants.

Compounds (µg/mL)		Must	SO_2_Sc **	NoSO_2_-Spont	NoSO_2_Sc
Phenolic acids	Gallic acid	0.10 b	0.05 a *	0.19 c	0.22 d
	Protocatechuic acid	0.16 a	0.17 a	0.19 a	0.31 b
	Caftaric acid	3.00 c	1.00 a	1.83 b	6.32 d
	Coutaric acid	2.20 b	1.92 a	2.38 c	2.98 d
	Caffeic acid	0.10 ab	0.08 a	0.13 b	0.15 b
	*p*-Coumaric acid	0.14 b	0.05 a	0.21 c	0.26 d
	Coumaric acid	0.11 a	0.13 a	0.13 a	0.14 a
	Ferulic acid	0.17 a	0.18 a	0.17 a	0.17 a
	**Sum**	**5.98 b**	**3.58 a**	**5.23 b**	**10.53 c**
Flavonols	Myricetin 3-*O*-glucoside	0.01 a	0.01 a	0.01 a	0.01 a
	Myricetin 3-*O*-rutinoside	0.01 a	0.01 a	0.03 b	0.03 b
	Quercetin 3-*O*-rutinoside	0.06 b	0.01 a	0.15 c	0.25 d
	Isorhamnetin 3-*O*-glucoside	0.03 b	0.01 a	0.04 b	0.12 c
	Quercetin 3-*O*-glucoside	0.12 b	0.03 a	0.38 c	0.55 d
	Dihydroquercetin 3-*O*-rhamnoside	0.02 a	0.03 a	0.02 a	0.05 b
	Quercetin 3-*O*-rhamnoside	0.20 b	0.05 a	0.70 c	1.19 d
	**Sum**	**0.45 b**	**0.15 a**	**1.32 c**	**2.20 d**
Flavan-3-ols	Procyanidin type B	1.60 a	2.92 b	2.93 b	3.60 c
	Procyanidin type B	0.85 a	1.36 b	1.42 b	1.79 c
	(+)-catechin	0.30 a	0.42 b	0.45 b	0.61 c
	Procyanidin type A	1.00 a	1.66 b	1.80 c	2.14 d
	Procyanidin type A	0.16 a	0.23 b	0.26 b	0.34 c
	(−)-epicatechin	2.10 a	3.16 b	4.04 c	5.44 d
	Epicatechin gallate	1.30 a	2.21 b	2.41 c	3.03 d
	**Sum**	**7.31 a**	**11.96 b**	**13.31 c**	**16.96 d**
Stilbenes	*Trans*-resveratrol	0.03 a	0.03 a	0.04 a	0.08 b
	*Cis*-resveratrol	0.18 a	0.26 b	0.28 b	0.35 c
	*Trans*-piceid	0.45 a	0.67 c	0.60 b	0.60 b
	*Cis*-piceid	0.25 a	0.27 a	0.42 b	0.56 c
	**Sum**	**0.91 a**	**1.23 a**	**1.36 ab**	**1.59 b**
**TOTAL**		**14.65 A**	**16.92 A**	**21.22 B**	**31.28 C**

Explanation: * Mean values followed by the same letter are not significantly different at *p* < 0.05 (Tukey’s HSD test). ** see explanation under Table 1.

**Table 5:** Identification parameters of polyphenolic compounds analysed by UPLC–PDA–MS/MS.

No.	Compound	RT *	[M-H]^−^	Fragment Ions	Absorbance Maxima
		(min.)	(m/z)	(m/z)	(nm)
*Phenolic acid*					
1	Gallic acid	1.47	169	125	272
2	Protocatechuic acid	2.25	153	109	308
3	Caftaric acid	2.49	311	179	328, 294
4	Coutaric acid	3.08	295	163	310
5	Caffeic acid	3.46	153	109	260, 294
6	*p*-Coumaric acid	4.39	163	119	308
7	Coumaric acid	4.82	163	119	310
8	Ferulic acid	4.92	193	134	323, 293
*Flavonols*					
1	Myricetin 3-*O*-glucoside	3.06	479	317	260, 353
2	Myricetin 3-*O*-rutinoside	3.53	625	463, 317	256, 356
3	Quercetin 3-*O*-rutinoside	3.73	609	447, 301	255, 355
4	Isorhamnetin 3-*O*-glucoside	4.26	477	315	254, 369
5	Quercetin 3-*O*-glucoside	4.32	463	301	253, 365
6	Dihydroquercetin 3-*O*-rhamnoside	4.67	449	303	253, 372
7	Quercetin 3-*O*-rhamnoside	4.99	447	301	254, 369
*Flavan-3-ols*					
1	Procyanidin type B	2.66	577	425, 285	280
2	Procyanidin type B	2.81	577	425, 285	276
3	(+)-catechin	3.01	289	-	280
4	Procyanidin type A	3.31	577	425, 285	279
5	Procyanidin type A	3.34	577	425, 285	280
6	(−)-epicatechin	3.68	289	-	280
7	Epicatechin gallate	4.06	441	289	279
*Stilbenes*					
1	*Trans*-resveratrol	6.24	227	185	327
2	*Cis*-resveratrol	7.42	227	143	327
3	*Trans*-piceid	4.75	389	227	327
4	*Cis*-piceid	5.94	389	227	327

Explanation: * RT, retention time.

## Data Availability

Data are contained within the article.
